# Effects of Fluroquinolones in Newly Diagnosed, Sputum-Positive Tuberculosis Therapy: A Systematic Review and Network Meta-Analysis

**DOI:** 10.1371/journal.pone.0145066

**Published:** 2015-12-15

**Authors:** Dandan Li, Tiansheng Wang, Su Shen, Sheng Cheng, Junxian Yu, Yang Zhang, Chao Zhang, Huilin Tang

**Affiliations:** 1 Department of Pharmacy, Beijing Friendship Hospital, Capital Medical University, Beijing, China; 2 Department of Pharmacy Administration and Clinical Pharmacy, School of Pharmaceutical Sciences, Peking University, Beijing, China; 3 Department of Pharmacy, Peking University Third Hospital, Beijing, China; Fundació Institut d’Investigació en Ciències de la Salut Germans Trias i Pujol, Universitat Autònoma de Barcelona, SPAIN

## Abstract

**Background:**

Tuberculosis is a major public health problem especially in developing countries, the comparative efficacy and safety of fluroquinolones (FQs) for adult patients with newly diagnosed, sputum-positive tuberculosis remains controversial. We aimed to investigate the benefits and risks of FQs-containing (addition/substitution) regimens in this population.

**Methods:**

A network meta-analysis was performed to compare FQs (C: ciprofloxacin; O: ofloxacin; Lo: levofloxacin; M: moxifloxacin; G: gatifloxacin) addition/substitution regimen with standard HRZE regimen (ie isoniazid, rifampicin, pyrazinamide and ethambutol) in newly diagnosed, sputum-positive tuberculosis. Medline, Embase and Cochrane Central Register of Controlled Trials were systematically searched, randomized trials with duration longer than 8 weeks were included. The primary outcome was week-8 sputum negativity, and secondary outcomes included treatment failure, serious adverse events and death from all cause.

**Results:**

Twelve studies comprising 6465 participants were included in the network meta-analysis. Löwenstein-Jensen culture method showed that HRZEM (OR 4.96, 95% CI 2.83–8.67), MRZE (OR 1.48, 95% CI 1.19–1.84) and HRZM (OR 1.32, 95% CI 1.08–1.62) had more sputum conversion than HRZE by the eighth week, whereas HRC (OR 0.39, 95% CI 0.19–0.77) and HRZO (OR 0.47, 95% CI 0.24–0.92) were worse than HRZE. Moxifloxacin-containing regimens showed more conversion than HRZE by liquid method at the end of two months. But by the end of treatment, FQs-containing regimens didn’t show superiority than HRZE on treatment failure. There were no significant differences between any regimens on other outcomes like serious adverse events and all-cause death.

**Conclusion:**

This comprehensive network meta-analysis showed that compared with HRZE, moxifloxacin-containing regimens could significantly increase sputum conversion by the eighth week for patients with newly diagnosed pulmonary tuberculosis while HRC and HRZO regimens were inferior. But all the FQs-containing regimens did not show superiority in other outcomes (such as treatment failure, serious adverse events and all-cause death). Thus, HRZE is still an effective regimen for this population. Although moxifloxacin-containing regimens have deomonstrated their potential, FQs-containing regimens should be used with great caution to avoid widespread FQs-resistance worldwide.

## Introduction

Tuberculosis (TB) is a major public health problem caused by *Mycobacterium tuberculosis bacteria*. According to the World Health Organization (WHO), 1.3 million patients among the 8.6 million cases died of TB in 2012, predominantly in developing countries [1]. While the first-line antituberculous regimen of isoniazid (H), rifampicin (R), pyrazinamide (Z), and ethambutol (E) (ie HRZE) recommended in the guideline has made breakthroughs in antituberculous therapy, multidrug-resistance (MDR), poor adherence and high rates of adverse events remain major challenges [1,2,3]. Thus, it is an urgent global health priority to develop least toxic regimens that could improve operational cure rates by shortening or simplifying treatment [4].

Fluroquinolones (FQs) have excellent early bactericidal activity and pharmacokinetic characteristics [5,6], results from mice experiments indicated that moxifloxacin (M) had greater bactericidal activity and more cure rates than streptomycin [7] or ethambutol [8]. Similar to moxifloxacin, gatifloxacin (G) appeared to have sufficient activity alone or in combination with ethambutol for treatment of TB in murine model [9]. However, there was no consensus about whether they can be used in practice for established first-line anti-TB therapy. An observational study in India first showed the possibilities to shorten the duration when ethambutol was replaced by ofloxacin[10]. Pilot studies substituting ciprofloxacin for pyrazinamide and ethambutol [11,12], ofloxaxin for ethambutol [13], or directly adding levofloxacin to the first-line regimen [14] did not show improvement on outcomes, ciprofloxacin was even associated with high rates of relapses [15,16]. On the other hand, studies substituting moxifloxacin for isoniazid [17,18,19] or ethambutol [13,18,20,21], gatifloxacin for ethambutol [13,22,23] or adding moxifloxacin to the HRZE regimen [21] demonstrated more sputum conversions by the eighth week even though Burman [24] did not show us the similar outcome in their study.

At the same time, it is worthy of attention that antibiotic improper use has been associated with the emergence of resistance to drugs for anti-TB therapy [25], which represents one of the most challenges to worldwide TB control[26,27]. FQs are currently one important class of antibiotics recommended for MDR cases (ie, resistant to at least rifampicin and isoniazid) [1], and rational use of these drug can help to protect FQs and prevent extensively drug-resistant TB (XDR-TB) [28]. Thus, it is very important to assess the efficacy and safety of available FQs in newly diagnosed TB without MDR, and weigh the benefits and risks we might meet before they are widely used.

Two previous meta-analyses evaluated FQs therapy for TB [15,29], but both studies solely pooled data from the limited direct evidence. The aim of this network meta-analysis was to assess the comparative efficacy and safety of FQs in adults with newly diagnosed, sputum-positive TB using both direct and indirect evidence [30] and discuss the future usage of FQs for newly diagnosed pulmonary TB patients.

## Materials and Methods

The systematic review and network meta-analysis was performed and reported according to PRISMA statement guidelines, and details were shown in S1 PRISMA Checklist.

### Search Strategy

We performed a systematic literature search for all relevant articles from inception through May 2015 in electronic databases including PubMed, Embase, Cochrane Central Register of Controlled Trials, ClinicalTrials.gov and the Web of Science. The search strategy was as followed:

1. tuberculosis
2. sputum positive
3. smear positive
4. positive sputum
5. 2 or 3 or 4
6. quinolone
7. fluoroquinolone
8. ciprofloxacin
9. ofloxacin
10. levofloxacin
11. moxifloxacin
12. gatifloxacin
13. or/6-12
14. 1 and 5 and 13

In addition, reference lists of included studies and reviews were also examined to check any eligible Randomized controlled trials (RCTs).

### Study Selection

RCTs fulfilling the predefined eligibility criteria were considered: (1) adult participants with age≥18; (2) newly diagnosed, sputum/smear-positive TB with previous treatment less than one month; (3) FQs addition/substitution regimens in the first-line antituberculous drugs; (4) studies with duration of more than eight weeks. Animal experiments or non-RCTs were excluded. We also excluded abstracts presented at conferences and from manufacturers’ websites because they did not provide sufficient information on patient characteristics, methods and relevant data for us to assess.

### Data Extraction and Quality Evaluation

Two reviewers independently extracted data into a standardized spreadsheet. Discrepancies were resolved by discussion, and a third reviewer was consulted when necessary. According to the Cochrane risk of bias tool, two reviewers independently assessed the quality of included study as “low”, “high” and “unclear” in the following six domains: selection, performance, detection, attrition, reporting and other bias [31].

### Outcomes and Data Synthesis

Normal anti-TB therapy included intensive phase and continuous phase. As we aimed to evaluate the efficacy and safety of addition/substitution of FQs during intensive phase of TB therapy, therapeutical differences during continuous phase were ignored. Due to limited number of eligible trials, we combined different dosing frequencies together and assumed they were clinically equivalent. For example, in Burman’s [24] four-arm RCT study, 5 day per week (d/w) HRZM and 3 d/w HRZM were combined to be HRZM regimen.

The primary outcome was sputum negative rates by the eighth week (week-8 sputum negativity). Secondary outcomes included treatment failure, serious adverse events (SAEs), and death from all-cause. In our analysis, treatment failure was defined as continued or recurrent positive sputum cultures (culture confirmed) and evaluated by the end of treatment; SAEs included grade 3 and higher adverse events including death according to the modified version of criteria from National Institute of Allergy and Infectious Diseases, Division of AIDS. SAE and death from all cause were assessed by the end of treatment and intensive phase separately.

### Statistical Analysis

We did two types of meta-analysis. First, we did standard pairwise meta-analysis with a random-effect model [32] by Revman 5.1 software for comparisons with at least two studies. Heterogeneity was assessed in these analyses with *I* ^*2*^ metric. Second, we did random-effect network meta-analysis by STATA (www.stata.com) using the ‘mvmeta’ [33] and self-programmed STATA routines assuming a common heterogeneity variable for all comparisons. Estimates from both primary and second outcomes were presented as odds ratios (OR) with 95% confidence intervals (CIs). 95% predictive intervals (PrIs) were also examined to capture their uncertainty and the magnitude of the heterogeneity in the network meta-analysis [34]. When the number of outcome events was zero, 0.5 was added based on the Haldane method [35]. To rank the treatments for an outcome, we used surface under the cumulative ranking (SUCRA) probabilities.

### Inconsistency

Consistency within every closed triangle or quadratic loop was investigated by loop-specific approach. During analysis, inconsistency factors and their 95% CIs were used to determine their compatibility with zero [36]. However, conclusions from this method should be explained cautiously because this approach cannot infer consistency of the entire network or identify the particular comparison that is problematic. We also investigated the global inconsistency in networks using the “design-by-treatment” interaction model.

### Publication Bias

Publication bias is a threat to the validity of meta-analysis, and funnel plot asymmetry is often used to assess the publication bias [37]. However, funnel plot test was inappropriate in this study because the outcomes were too disperse, and there were less than four studies involved in all direct comparisons even though the number of included trials were larger than ten [38]. We did not downgrade our confidence in the evidence of publication bias because of the comprehensive search strategy we followed.

## Results

**Fig 1:**
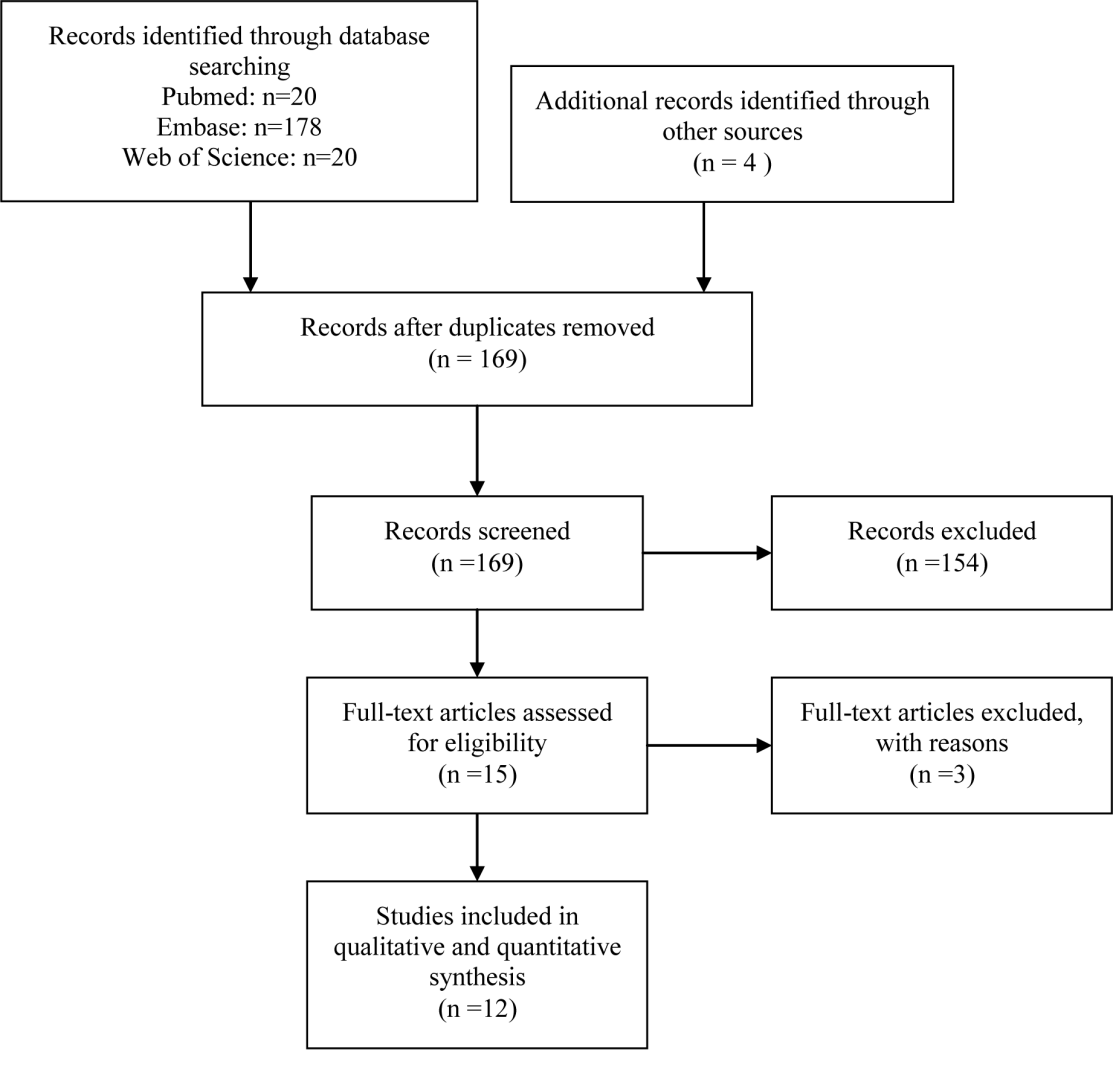
PRISMA flow chart of articles included in this network meta-analysis.

We identified 222 citations, of which 53 were duplicates, leaving 169 potentially relevant citations. After screening for title and abstract, 154 citations were excluded due to non-randomized studies, short duration less than eight weeks or irrelevant diseases and intervention. 15 full texts were retrieved for further assessment, and finally 12 RCTs involving 6,465 patients were included in the baseline characteristic analysis (Fig 1).

### Study Characteristics and Study Quality

S1 Table illustrated the characteristics of included studies. These studies were conducted in Africa [11–13,17–19,22,24], North America [14,18,19,24], South America [19,20], Europe[19] and Asia [18,21,23]. Regimens compared in this review were HRZE, MRZE, HRZM, HRZG, HRZO, HRC, HRZEM and HRZELo. According to S1 Table, five studies cultured their samples on both Löwenstein-Jensen solid medium and liquid medium [13,17–20], follow-up time ranged from 2 months to 30 months after randomization. During the data collection, a group of participants in the study of El-Sadr were excluded [14], because participants younger than 18 years old (older than 13 years old) were included and no control group was set. Besides, Kennedy reported two independent studies in one publication [12], the first one was excluded because it was designed to evaluate the early bactericidal activity of ciprofloxacin and isoniazid within seven days and didn’t assess the outcomes we cared about.

**Fig 2:**
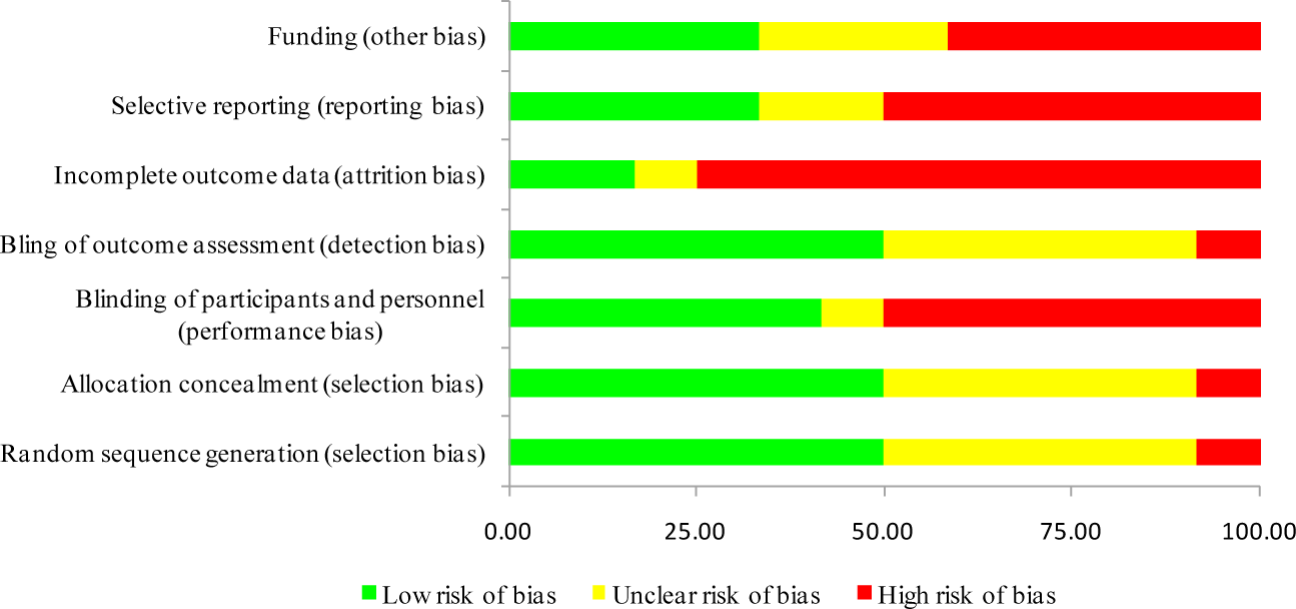
Risk of bias summary graph. Review authors’ judgments (Low, Unclear and High) for each risk of bias item shown as percentages across all included studies.

The risks of bias for each study were shown in S2 Table. Studies were seemed as high risk of incomplete outcome if they reported only part of the outcomes we assessed. When there were more than 10% missing from follow-up analyses or imbalanced numbers across intervention groups for loss to follow-up, studies were also deemed to be high risk for incomplete outcome data. Low risk of other bias meant that none of the funding agencies was involved in any aspects of the study. The summary graph of risk of bias was shown in Fig 2.

### Network and Pairwise Meta-analysis for Efficacy and Safety

**Fig 3:**
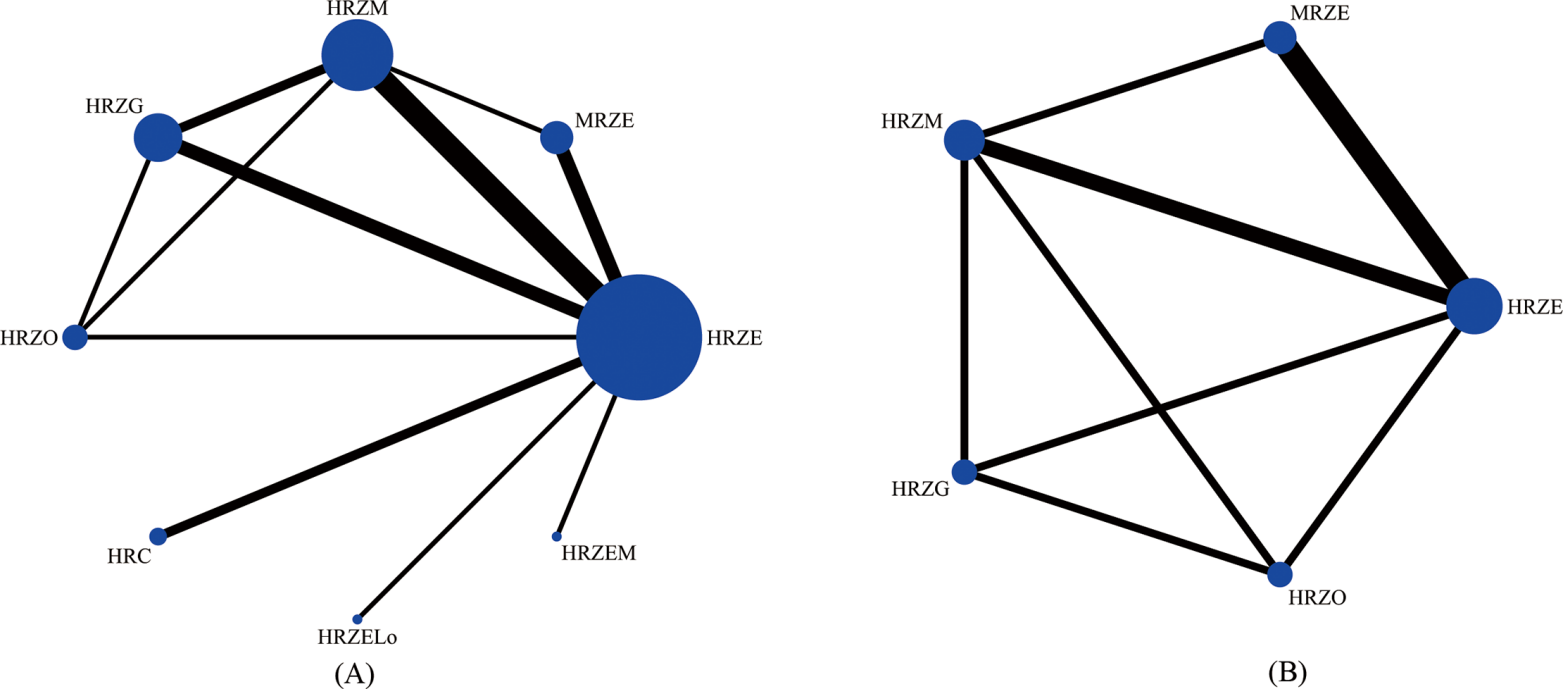
Network of possible interventions for week-8 sputum negativity. (A) Löwenstein-Jensen solid method; (B) liquid method.

Networks of eligible comparisons for week-8 sputum negativity were presented in Fig 3, showing predominantly pairwise comparisons of different regimens with HRZE. Networks for secondary outcomes (S1 Fig) showed a similar preponderance and all these trials considered HRZE regimen as control group.

Data for direct comparisons and network estimates for both primary and secondary outcomes were shown in S3 and S4 Tables. We also ranked the comparative effects of all drugs against HRZE with SUCRA probabilities.

#### Primary Outcome: Week-8 Sputum Negativity

**Fig 4:**
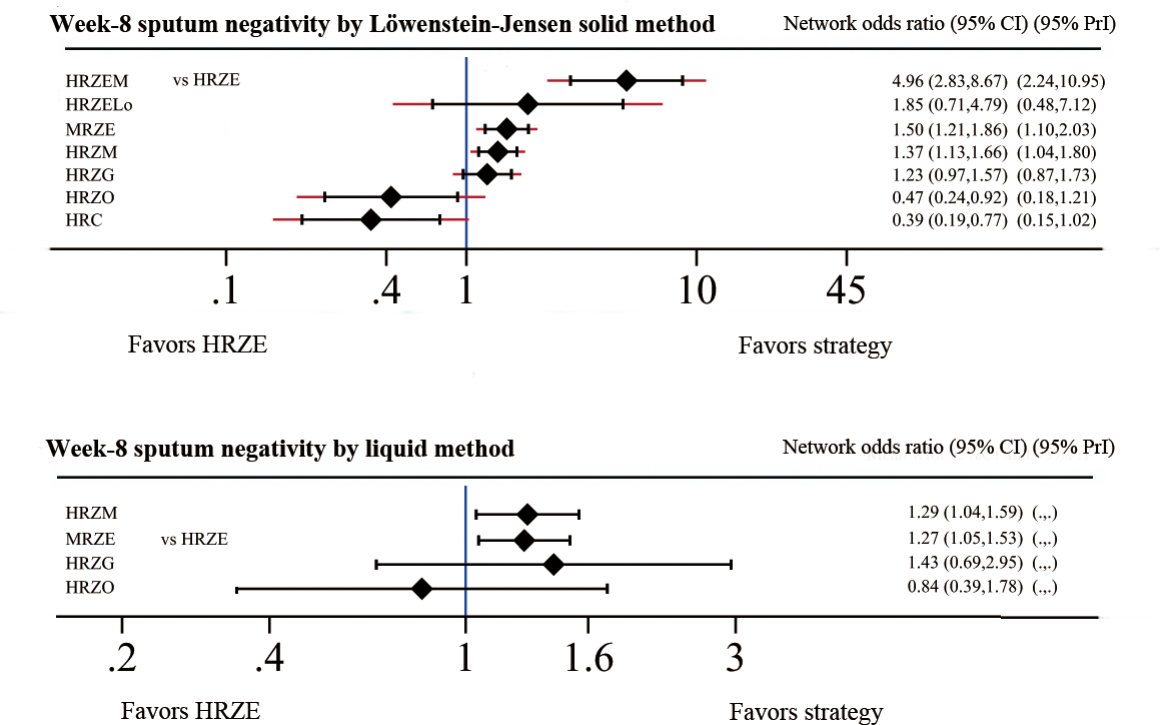
Network meta-analysis of strategies compared with HRZE for primary outcomes.

Week-8 sputum negativity by Löwenstein-Jensen solid culture method was reported in all the included studies. HRZEM (OR 4.96, 95% CI 2.83–8.67), MRZE (OR 1.48, 95% CI 1.19–1.84) and HRZM (OR 1.32, 95% CI 1.08–1.62) had more sputum conversion than HRZE regimen, HRC (OR 0.39, 95% CI 0.19–0.77) and HRZO (OR 0.47, 95% CI 0.24–0.92) were worse than HRZE at week-8 sputum negativity (Fig 4). OR estimates ranged from 4.96 (95% CI 2.83–8.67) for the highest ranked treatment strategy (HRZEM) to 0.39 (95% CI 0.19–0.77) for the lowest ranked regimen (HRC).

Even though fewer studies provide data on liquid medium, the results of network meta-analysis were similar with those of solid medium (Fig 4). HRZM (OR 1.29, 95% CI 1.04–1.59) and MRZE (OR 1.27, 95% CI 1.07–1.50) regimens had more conversion than HRZE, but HRZG ranked the highest by 1.43 (95% CI 0.69–2.95). No other statistical difference was detected.

The results of direct comparison including at least two trials were shown in S3 Table. There was more sputum conversion in MRZE (OR 1.46, 95% CI 1.17–1.82), HRZM (OR 1.50, 95% CI 1.11–2.03) and HRZG (OR 1.27, 95% CI 1.00–1.63). HRZEM (OR 4.96, 95% CI 2.83–8.67) was not included in the table because only one study was included. MRZE (OR 1.27, 95% CI 1.06 to 1.53) and HRZM (OR 1.25, 95% CI 1.00 to 1.55) also showed more conversion than HRZE based on results from liquid medium.

#### Second Outcome: Treatment Failure by the end of treatment

**Fig 5:**
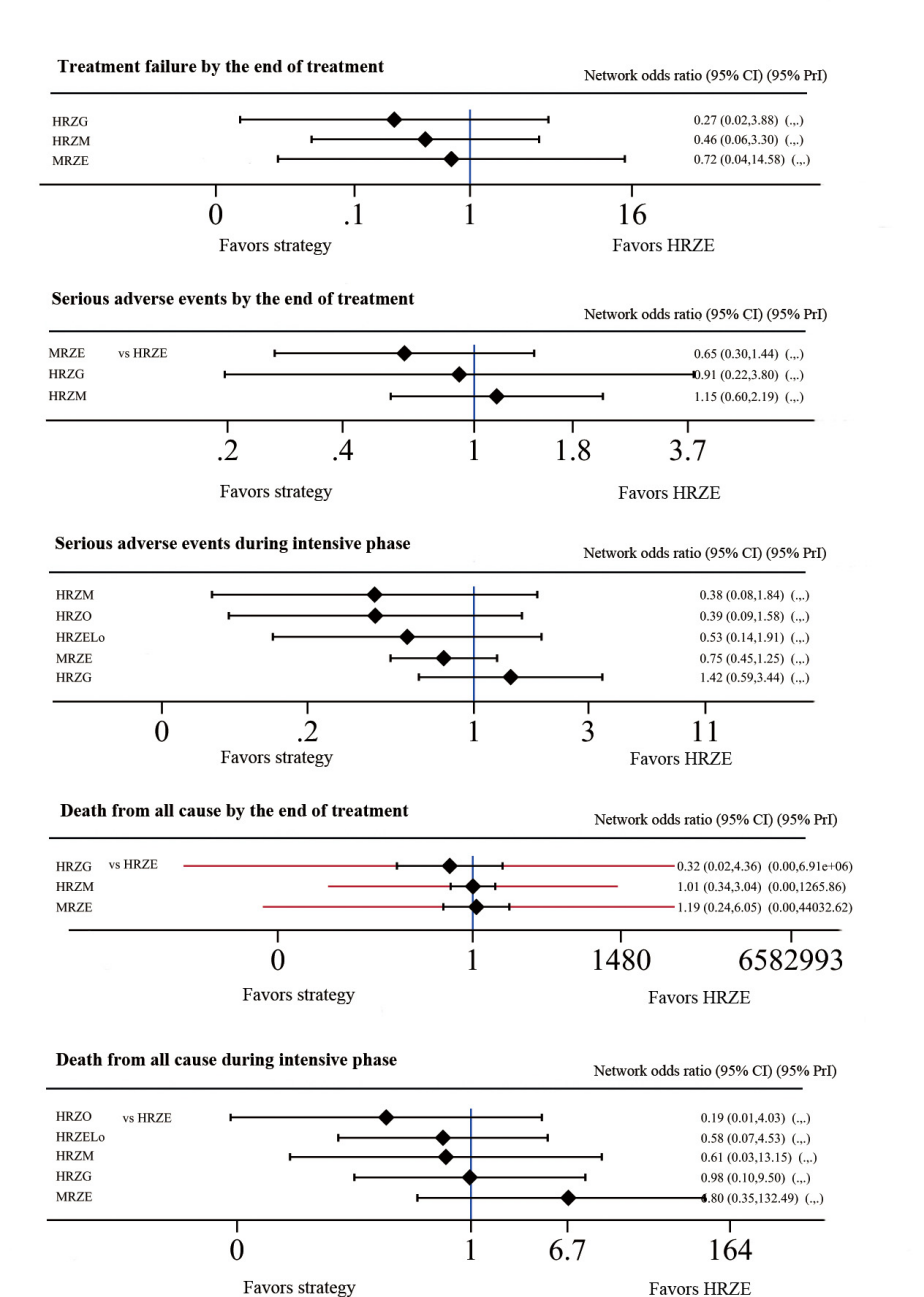
Network meta-analysis of strategies compared with HRZE for second outcomes.

Fig 5 presented estimated effects of drug regimens on treatment failure by the end of treatment. MRZE, HRZM and HRZG regimens were superior to HRZE, but the differences were not statistically significant (OR 0.72, 95% CI 0.04–14.58; OR 0.46 95% CI 0.06–3.30; and OR 0.27, 95% CI 0.02–3.88, respectively). Among them, HRZG (OR 0.27, 95% CI 0.02–3.88) got the highest SUCRA score.

Direct comparison also indicated no statistical difference among these regimens involved (S3 Table).

#### Secondary Outcome: Serious Adverse Events

According to included trials, the most common SAEs were gastrointestinal disorders, neurological disorders, skin and appendages disorders, cutaneous disorders, urinary system disorders and so on. We compared the incidence of SAEs but no statistical differences were found among them by the end of treatment and during the two-month intensive phase. And the detailed results were demonstrated in Fig 5. Jawaha [23] reported more drug-related adverse events for HRZM and HRZG regimens, but these data were excluded in our analysis because little information was provided regarding the grade of these adverse events.

Pairwise meta-analysis (S3 Table) showed that MRZE (OR 0.87, 95% CI 0.60–1.25) and HRZM (OR 0.83, 95% CI 0.55–1.26) seemed better than HRZE by the end of treatment, but no statistical differences existed.

#### Secondary Outcome: Death from All Cause

Network meta-analysis results of death from all cause were demonstrated in Fig 5. By the end of treatment (at most 30 d after the treatment) and during intensive phase, no significant difference was found. According to the cumulative ranking curve, HRZG had the greatest likelihood to cause the least all-cause deaths by the end of treatment and HRZO got the highest score during intensive phase.

Based on the pairwise meta-analysis result, HRZM might cause less death than HRZE (OR 0.73, 95% CI 0.25–2.11 for the end of treatment and OR 0.74, 95% CI 0.16–3.36 for intensive phase), but there was no statistical difference.

#### Inconsistency Check

In the network meta-analysis, no statistical heterogeneity was seen for all the outcomes (S5 Table). Global inconsistency was not noted within any network (S6 Table). In pairwise comparison for the primary and secondary outcomes, no statistical heterogeneity was observed in most of these comparisons (S3 Table) except for HRZM vs HRZE, HRC vs HRZE in week-8 sputum negativity by solid medium, and MRZE vs HRZE in SAE by the end of treatment. But these results for inconsistency might be imprecise because of the sparse data in the limited number of trials.

## Discussion

To our knowledge, this is the first systematic review to evaluate the efficacy and safety of FQs for newly diagnosed pulmonary TB by NMA method. Different from previous meta-analysis [29], this study indicated that HRZEM, MRZE and HRZM regimens might increase the week-8 sputum negativity, but HRC (OR 0.39, 95% CI 0.19–0.77) and HRZO (OR 0.47, 95% CI 0.24–0.92) were worse than HRZE. No significant differences were detected in other outcomes of our interest including treatment failure by the end of treatment.

Consistent with good early bactericidal effects [5,39], this network meta-analysis indicated that moxifloxacin-containing regimens could increase conversion rate at week 8. Shorter time to culture conversion can also confirm the early sterilizing activity of moxifloxacin: compared with HRZE, the hazard ratio of time to culture conversion was 1.73 to HRZM in Rustomjee’s study [13]. However, culture-confirmed treatment failure in our meta-analysis did not show meaningful advantage between FQs-containing regimens and HRZE by the end of treatment. Prevention of relapse was one of the major aims of anti-TB therapy issued in the guideline [40], moxifloxacin-containing regimens should be able to reduce relapse rates based on the culture results because previous study had suggested a strong relationship between relapse and week-8 sputum negativity [41]. Yet, three out of four moxifloxacin-involving studies [17,18,22] showed that moxifloxacin had a higher relapse than HRZE despite of the variation of continuation drugs and follow-up time.

These disparities might be due to the insufficient drug exposure (daily and total dose). The dosage of moxifloxacin (400 mg) was fixed and not adjusted on the basis of body weight in these studies. Total exposure to the FQs-containing regimen might have been insufficient for sustained sterilization of the respiratory tract, especially in patients with cavitation or a higher BMI. This was indirectly supported by a population pharmacokinetic model [42], which predicted that only 62% of the study population would have a target ratio of area under the curve to minimal inhibitory concentration. Another reason could be the present of non-replicating tubercle bacilli [43], which took longer to initiate growth than their replicating counterparts, and were tolerant to the bactericidal action of antibiotics [44]. FQs act by inhibiting DNA gyrase and topoisomerase IV involved in bacterial DNA synthesis, thereby enable these agents to be bactericidal [45]. While ethambutol was introduced as a synthetic bacteriostatic agent because it could disrupt bacterial metabolism by interfering with essential metal-containing enzyme systems, causing arrest of cell multiplication and cell death [46]. Substitution of ethambutol by bactericidal FQs might undermine the delayed anti-TB effect. At the same time, this heterogeneity might come from imprecision because large number of patients lost during follow-up and occurrence of treatment failure and relapse was sparse.

Our analysis results indicated no statistical difference between regimens on SAEs. Although most common serious adverse events caused by FQs were hyperglycemic and QT prolongation, we were not able to evaluate risk of anti-TB related SAEs because they were not exhaustively reported and the incidence was low. Subgroup analysis of week-8 sputum negativity for HIV patients was not feasible because majority of included studies did not report sputum conversion of this subpopulation. Though El-Sadr [14] included only HIV-positive patients in their study and Burman [24] reported the conversion rate in patients with HIV, but different experimental regimens from two studies couldn’t be combined. HIV serostatus might be unassociated with culture conversion at week 8 because it happened in 72% of HIV-positive patients and 71% of HIV-negative patients according to Burman [24]. In addition, Dorman [19] suggested that HIV was not a factor associated with negative sputum culture by the eighth week of treatment (OR 1.52, 95% CI 0.75–3.08, P 0.24). We were unable to evaluate the relapse rate of different regimens either because the follow-up time varies in included studies, combination of incomplete data might cause misleading results.

According to our results, FQs-containing regimens did not show superiority than the standard regimen. This has several implications for practice and research. First of all, considering that the presence of nonreplicating tubercle bacilli might affect the use of FQs, it might be better to identify this subpopulation in sputum prior to commencement of therapy [43]. Secondly, the discrepancy between week-8 sputum negativity and treatment failure as well as relapse refutes the predictability of culture conversion for long-term outcomes. Thus, their correlation should be further examined. Thirdly, numbers of positive cultures by the Löwenstein-Jensen and liquid method were heterogeneous in some included studies [18,47], we recommend future studies to report culture results separately using both Löwenstein-Jensen solid and liquid method. At last, HRZE regimen is still an effective regimen in newly diagnosed, sputum negativity TB. FQs are key components of current MDR-TB treatment regimens [48,49] for their higher mutant prevention concentration [50], and better pharmacokinetics [51]. XDR was defined as MDR-TB plus resistance to any fluoroquinolone and at least one injectable second-line drug (i.e., amikacin, kanamycin or capreomycin). Development of XDR would lead to longer duration of treatment, higher risk of adverse events and more unsuccessful treatment outcomes [52,53]. Thus, FQs should be used cautiously to avoid further resistance especially in areas with high incidence of MDR like China, India and Russia [1]. FQs-containing regimens shouldn’t be considered as the first choice for newly diagnosed TB patients according to the results of this meta-analysis.

Our study has several strengths. Firstly, our network meta-analysis allowed comparison of all available FQs-related anti-TB therapy using both direct and indirect evidence. Secondly, our study analyzed the results based on solid and liquid medium separately to reduce heterogeneity among different culture methods. Thirdly, we applied mITT population instead of ITT population by the previous review [39]. The ITT principle implies that patients were analyzed according to their original allocatio regardless of the treatment they actually received, while mITT analysis allows authors to further exclude patients betraying original design but receiving intervention or control [54]. As most of the included studies had identified cases with negative-sputum, drug-resistance which deviated from the original study design, using mITT population will provide a more accurate result by excluding these cases. Fourthly, the trial sites were geographically diverse especially in low-income and middle-income countries, which meant that the results were likely to be applicable to situations where the burden of TB is high.

We acknowledge the following limitations to our work. Firstly, data used in the network meta-analysis was based on a limited number of studies, which may result in imprecision due to sparse data [55]. For example, regimens like HRZELo [14], HRZO [13] and HRZEM [21] were only assessed separately in one clinical trial, bias in this trial might affect the accuracy of our result. Secondly, there was heterogeneity between trial populations which might affect the accuracy of our results. For example, patients from Jawahar’s [23] and Velayutham’s study [21] were all HIV negative, while El-Sadr [14] included only HIV positive patients. However, “loop-specific” consistency and “design-by-treatment” global inconsistency tests didn’t show any inconsistency between all participants from different head to head comparisons. This also indicated the shortage of meta-analysis method. Thirdly, we didn’t include unpublished, premarketing studies or studies with only abstract, which might lead to reporting bias.

## Conclusion

This comprehensive network meta-analysis showed that compared with HRZE, moxifloxacin-containing regimens might significantly increase sputum conversion by the eighth week for patients with newly diagnosed pulmonary TB, while HRC and HRZO regimens were inferior. But all the FQs-containing regimens didn’t show superiority at other aspects like treatment failure, SAE and death. Thus, FQs-containing regimens should be used with caution considering the widespread resistance worldwide.

## Supporting Information

S1 PRISMA Checklist (DOC)

S1 Table Baseline characteristics of included study. (DOC)

S2 Table Risk of bias summary. Review authors’ judgments about each risk of bias item for each included study. Each study is shown in the vertical axis and the corresponding risk of bias for each domain adjudicated by two authors is shown by colored circles within the grid.
(DOC)

S3 Table Results of pairwise meta-analyses and heterogeneity of regimens with at least two trials involved. (DOC)

S4 Table Network results of second outcomes estimated by odds ratios (95% confidence intervals). (A) Week-8 sputum negativity by Löwenstein-Jensen solid method; (B) Week-8 sputum negativity by liquid method; (C) Treatment failure by the end of treatment; (D) Serious adverse events by the end of treatment; (E) Serious adverse events during intensive phase; (F) Death from all cause by the end of treatment; (G) Death from all cause during intensive phase.
(DOC)

S5 Table Evaluation of consistency using “loop specific” approach. (DOC)

S6 Table Assessment of global inconsistency in networks using the “design-by-treatment” interaction model. (DOC)

S1 Fig Network of possible interventions for second outcomes. (A) Treatment failure by the end of treatment; (B) Serious adverse events by the end of treatment; (C) Serious adverse events during intensive phase; (D) Death from all cause by the end of treatment; (E) Death from all cause during intensive phase.
(DOC)
