# Supplementary material for: Effects of Fluroquinolones in Newly Diagnosed, Sputum-Positive Tuberculosis Therapy: A Systematic Review and Network Meta-Analysis
Source: PLoS One. 2015 Dec 15;10(12):e0145066. doi: 10.1371/journal.pone.0145066 (PMC4682926; doi:10.1371/journal.pone.0145066)
Supplement: S1 Table — (DOC) [file pone.0145066.s002.doc]

**S1Table. Baseline characteristics of included study**

| **Author** | **Ref.** | **Journal** | **Country of study** | **Regimens compared** | **Inclusion Criteria** | **Drug resistance condition** | **Culture medium** | **Participants** | **Male sex no.(%)** | **Age (mean±SD)** | **weight (mean±SD)** | **HIV-positive no.(%)** | **CD4 count median (IQR)** | **cavitation no.(%)** | **Duration (month)** | **Follow-up (month)** |
| --- | --- | --- | --- | --- | --- | --- | --- | --- | --- | --- | --- | --- | --- | --- | --- | --- |
| Burman et al. 2006 | 24 | Am. J. Respir. Crit. Care. Med. | Africa, North America | HRZE; HRZM | Age ≥18y, sputum-positive TB, less than 7d ﬂuoroquinolone antibiotic or anti-TB treatment within 6m. | Susceptible to R, Z, M. Resistant to H: 9.0%. | LJ solid medium | 277 | 186 (67) | ------ | ------ | 60(28) | ------ | ------ | 6 | 6 |
| Conde et al. 2009 | 20 | Lancet | Brazil | HRZE; HRZM | Age ≥18y, drug sensitive, smear-positive TB without previous treatment. | Susceptible to H, R, E. | LJ solid and liquid media | 146 | 91 (62) | 34.1±11.9 | 56.2±9.9 | 5(3) | ------ | 101(69) | 6 | 18 |
| Dorman et al. 2009 | 19 | Am. J. Respir. Crit. Care. Med. | North America, Brazil, South Africa, Spain, Uganda | HRZE; MRZE | Age ≥18 years, sputum-positive TB, without previous treatment. | Susceptible to H, R, Z, M. | LJ solid and liquid media | 381 | 275(72) | 31 (26, 40)^a^ | ------ | 41(11) | 221(112, 334) | 283(74) | 6 | 6 |
| El-Sadr et al. 1998 | 14 | Clin. Infect. Dis. | United States | HRZE; HRZELo | Age ≥18 years with HIV， culture-conﬁrmed TB, less than 1 month anti-TB chemotherapy. | Susceptible to H, R. | LJ solid meidum | 101 | 271(27) | 40 | ------ | 1015(100) | ------ | ------ | 6, 9 | 26.2^b^ |
| Gillespie et al. 2014 | 18 | N. Engl. J. Med. | South Africa, India, Tanzania, Kenya, Thailand, Malaysia, Zambia, China, Mexico | HRZE; HRZM; MRZE | Age ≥18y, smear-positive TB, previously untreated, susceptible to R and FQs. | Susceptible to R, M. Resistant to H: 7%; Z 2%. | LJ solid and liquid media | 1674 | 1166(70) | ------ | ------ | 123(7) | ------ | 1170(70) | 4, 6 | 18 |
| Jawahar et al 2013. | 23 | PLoS One | South India | HRZE; HRZM; HRZG | Age≥18y, newly diagnosed, sputum-positive HIV-negative TB patients. | Susceptible to H, R, E, O: 90.9%. Resistant to any drug: 9.1%. | LJ solid media | 416 | 108 (26) | ------ | 43.6 | 0 | ------ | ------ | 4, 6 | 28, 30m |
| Jindani et al. 2014 | 17 | N. Engl. J. Med. | South Africa, Zimbabwe, Botswana, and Zambia | HRZE; MRZE | Age>18y，weight ≥35 kg, smear-positive, less than 1 month anti-TB chemotherapy. | Susceptible to H, R, M | LJ solid and liquid media | 593 | 379(64) | ------ | ------ | 158(27) | 314(253, 441) | ------ | 4, 6 | 12-18 |
| Kennedy et al. 1993 | 12 | Am Rev Respir Dis | Northern Tanzania | HRZE, HRC | Age ≥18 years, newly diagnosed, smear-positive TB. | Susceptible to H, R, E, C. | LJ solid media | 20 | 10 (50) | 30.3 | ------ | 40% | ------ | ------ | 2 | 2 |
| Kennedy et al. 1996 | 11 | Clin. Infect. Dis. | Tanzania | HRZE; HRC | Age ≥18 y, newly diagnosed, smear-positive TB. | Susceptible to H, R, Z, E, C | LJ solid meidum | 168 | 115(68) | 34.8 | 49.3±7.66 | 58(35) | ------ | 107(64) | 6 | 12 |
| Merle et al. 2014 | 22 | N. Engl. J. Med. | Benin, Guinea, Kenya, Senegal, Durban | HRZE; HRZG | Age: 18-65y, newly diagnosed sputum positive TB. | Susceptible to R, Z, E, G. Resistant to H: 5.5%. | LJ solid meida | 1692 | 1230(73) | ------ | ------ | 304(18) | ------ | 857(51) | 4, 6 | 28, 30 |
| Rustomjee et al. 2008 | 13 | Int. J. Tuberc. Lung. Dis. | KwaZulu Natal | HRZE; HRZG; HRZM; HRZO | Age: 18-65y, weight: 38-80 kg, newly diagnosed, smear-positive TB. | Susceptible to R. | LJ solid and liquid midia | 217 | 145(67) | 31.5±9.1 | 55.2±7.9 | 127(59) | 246.7(100, 348) | 204(94) | 6 | 6 |
| Velayutham et al. 2014 | 21 | Clin Infect Dis | South India | HRZE; HRZEM | Age ≥18 y, smear-positive TB without HIV, less than 1 month treatment. | Susceptible to H, R, E, O: 86.4%. Resistant to any drug: 13.6%. | LJ solid media | 780 | 198 (25) | ------ | ------ | 0 | ------ | ------ | 3, 4, 6 | 27, 28, 30 |
| H: isoniazid; R: rifampicin; Z: pyrazinamide; E: ethambutol; C: ciprofloxacin; O: ofloxacin; Lo: levofloxacin; M: modifloxacin; G: gatifloxacin; FQs: fluroquinolones.  LJ: Löwenstein-Jensen  IQR = interquartile range  ^a^: results expressed as median (IQR)  ^b^: median follow-up time | | | | | | | | | | | | | | | | |
