# Supplementary material for: Effects of Fluroquinolones in Newly Diagnosed, Sputum-Positive Tuberculosis Therapy: A Systematic Review and Network Meta-Analysis
Source: PLoS One. 2015 Dec 15;10(12):e0145066. doi: 10.1371/journal.pone.0145066 (PMC4682926; doi:10.1371/journal.pone.0145066)
Supplement: S2 Table — Review authors’ judgments about each risk of bias item for each included study. Each study is shown in the vertical axis and the corresponding risk of bias for each domain adjudicated by two authors is shown by colored circles within the grid. (DOC) [file pone.0145066.s003.doc]

**S2 Table. Risk of bias summary.** Review authors’ judgments about each risk of bias item for each included study. Each study is shown in the vertical axis and the corresponding risk of bias for each domain adjudicated by two authors is shown by colored circles within the grid.

| **Authors** | **Ref.** | **Selection bias** | | **Performance bias** | **Detection bias** | **Attrition bias** | **Reporting bias** | **Other bias** |
| --- | --- | --- | --- | --- | --- | --- | --- | --- |
|  |  | **Random sequence generation** | **Allocation concealment** | **Blinding of participants and personnel** | **Bling of outcome assessment** | **Incomplete outcome data** | **Selective reporting** | **Funding** |
| Burman et al. 2006 | 24 |  |  |  |  |  |  |  |
| Conde et al. 2009 | 20 |  |  |  |  |  |  |  |
| Dorman et al. 2009 | 19 |  |  |  |  |  |  |  |
| El-Sadr et al. 1998 | 14 |  |  |  |  |  |  |  |
| Gillespie et al. 2014 | 18 |  |  |  |  |  |  |  |
| Jawahar et al 2013. | 23 |  |  |  |  |  |  |  |
| Jindani et al. 2014 | 17 |  |  |  |  |  |  |  |
| Kennedy et al. 1993 | 12 |  |  |  |  |  |  |  |
| Kennedy et al. 1996 | 11 |  |  |  |  |  |  |  |
| Merle et al. 2014 | 22 |  |  |  |  |  |  |  |
| Rustomjee et al. 2008 | 13 |  |  |  |  |  |  |  |
| Velayutham et al. 2014 | 21 |  |  |  |  |  |  |  |
| Green (+) = low risk, yellow (?) = unclear risk, red (-) = high risk. Other biases include one or more of: sponsor involved in study design, analysis, or authorship; imbalance between treatment comparisons. | | | | | | | | |
