# Supplementary material for: Effects of Fluroquinolones in Newly Diagnosed, Sputum-Positive Tuberculosis Therapy: A Systematic Review and Network Meta-Analysis
Source: PLoS One. 2015 Dec 15;10(12):e0145066. doi: 10.1371/journal.pone.0145066 (PMC4682926; doi:10.1371/journal.pone.0145066)
Supplement: S3 Table — (DOC) [file pone.0145066.s004.doc]

**S3 Table. Results of pairwise meta-analyses and heterogeneity of regimens with at least two trials involved.**

| **Comparisons** | | **Pairwise meta-analysis**  **OR (95% CI)** | **No. of**  **participants** | **No. of**  **trials** | **No. of events** | **Heterogeneity I^2^** |
| --- | --- | --- | --- | --- | --- | --- |
| **Week-8 sputum negativity by solid medium** | | | | | | |
| MRZE | HRZE | 1.46 (1.17, 1.82) | 1863 | 3 | 1431 | 0.0% |
| HRZM | HRZE | 1.50 (1.11, 2.03) | 2010 | 5 | 1421 | 36%^*^ |
| HRZG | HRZE | 1.27 (1.00, 1.63) | 1875 | 3 | 1561 | 0.0% |
| HRC | HRZE | 0.38 (0.18, 0.82) | 188 | 2 | 140 | 2%^*^ |
| HRZG | HRZM | 0.70 (0.39, 1.26) | 341 | 2 | 284 | 0.0% |
| **Week-8 sputum negativity by liquid medium** | | | | | | |
| MRZE | HRZE | 1.27 (1.06, 1.53) | 1972 | 3 | 1108 | 0.0% |
| HRZM | HRZE | 1.25 (1.00, 1.55) | 1304 | 2 | 609 | 0.0% |
| **Treatment failure by the end of treatment** | | | | | | |
| MRZE | HRZE | 0.40 (0.09, 1.73) | 1699 | 2 | 8 | 0.0% |
| HRZM | HRZE | 0.81 (0.25, 2.62) | 1400 | 2 | 12 | 0.0% |
| HRZG | HRZE | 0.75 (0.38, 1.47) | 1651 | 2 | 35 | 0.0% |
| **Serious adverse events by the end of treatment** | | | | | | |
| MRZE | HRZE | 0.87 (0.60, 1.25) | 2102 | 2 | 127 | 10%^*^ |
| HRZM | HRZE | 0.83 (0.55, 1.26) | 1440 | 2 | 95 | 0.0% |
| **Serious adverse events during intensive treatment phase** | | | | | | |
| HRZM | HRZE | 1.06 (0.52, 2.15) | 585 | 3 | 33 | 0.0% |
| **Death from all cause by the end of treatment** | | | | | | |
| MRZE | HRZE | 1.10 (0.50, 2.45) | 2521 | 3 | 30 | 0.0% |
| HRZM | HRZE | 0.73 (0.25, 2.11) | 1440 | 2 | 14 | 0.0% |
| **Death from all cause during intensive phase** | | | | | | |
| HRZM | HRZE | 0.74 (0.16, 3.36) | 585 | 3 | 5 | 0.0% |

^*^: results with statistical heterogeneity.
