# Supplementary material for: Effects of Fluroquinolones in Newly Diagnosed, Sputum-Positive Tuberculosis Therapy: A Systematic Review and Network Meta-Analysis
Source: PLoS One. 2015 Dec 15;10(12):e0145066. doi: 10.1371/journal.pone.0145066 (PMC4682926; doi:10.1371/journal.pone.0145066)
Supplement: S4 Table — (A) Week-8 sputum negativity by Löwenstein-Jensen solid method; (B) Week-8 sputum negativity by liquid method; (C) Treatment failure by the end of treatment; (D) Serious adverse events by the end of treatment; (E) Serious adverse events during intensive phase; (F) Death from all cause by the end of treatment; (G) Death from all cause during intensive phase. (DOC) [file pone.0145066.s005.doc]

**S4 Table. Network results of second outcomes estimated by odds ratios (95% confidence intervals).** Results underlined are statistically different.

1. Week-8 sputum negativity by Löwenstein-Jensen solid method

| HRZEM |  | | | | | | |
| --- | --- | --- | --- | --- | --- | --- | --- |
| 2.68(0.89-8.09) | HRZELo |  | | | | | |
| 12.86(5.29-31.25) | 4.80(1.48-15.53) | HRC |  | | | | |
| 10.51(4.41-25.05) | 3.92(1.23-12.51) | 0.82(0.31-2.13) | HRZO |  | | | |
| 4.03(2.19-7.40) | 1.50(0.56-4.01) | 0.31(0.15-0.65) | 0.38(0.19-0.75) | HRZG |  | | |
| 3.62(2.00-6.54) | 1.35(0.51-3.56) | 0.28(0.14-0.58) | 0.34(0.18-0.68) | 0.90(0.67-1.21) | HRZM |  | |
| 3.31(1.82-6.03) | 1.23(0.46-3.27) | 0.26(0.12-0.53) | 0.31(0.16-0.63) | 0.82(0.60-1.13) | 0.91(0.73-1.15) | MRZE |  |
| 4.96(2.83-8.67) | 1.85(0.71-4.79) | 0.39(0.19-0.77) | 0.47(0.24-0.92) | 1.23(0.97-1.57) | 1.37(1.13-1.66) | 1.50(1.21-1.86) | HRZE |

1. Week-8 sputum negativity by liquid method

| HRZO |  | | | |
| --- | --- | --- | --- | --- |
| 0.58(0.25-1.35) | HRZG |  | | |
| 0.65(0.31-1.39) | 1.11(0.54-2.30) | HRZM |  | |
| 0.66(0.31-1.42) | 1.13(0.54-2.35) | 1.01(0.82-1.25) | MRZE |  |
| 0.84(0.439-1.78) | 1.43(0.69-2.95) | 1.29(1.04-1.59) | 1.27(1.07-1.50) | HRZE |

1. Treatment failure by the end of treatment

| HRC |  | | | |
| --- | --- | --- | --- | --- |
| 1.13(0.02-76.65) | HRZG |  | | |
| 0.80(0.01-54.04) | 0.71(0.08-5.98) | HRZM |  | |
| 2.27(0.03-184.37) | 2.00(0.17-23.99) | 2.83(0.24-33.60) | MRZE |  |
| 1.30(0.29-5.86) | 0.27(0.0.02-3.88) | 0.46 (0.06-3.30) | 0.72(0.04-14.58) | HRZE |

1. Serious adverse events by the end of treatment

| HRZG |  | | |
| --- | --- | --- | --- |
| 1.03(0.49-2.17) | HRZM |  | |
| 0.75(0.31-1.82) | 0.73(0.33-1.59) | MRZE |  |
| 0.91(0.22-3.80) | 1.15(0.60-2.19) | 0.65(0.30-1.44) | HRZE |

1. Serious adverse events during intensive phase

| HRZELo |  | | | | |
| --- | --- | --- | --- | --- | --- |
| 1.56(0.33-7.40) | HRZO |  | | | |
| 2.13(0.41-11.11) | 1.36(0.29-6.38) | HRZG |  | | |
| 0.58(0.17-1.99) | 0.37(0.08-1.77) | 0.27(0.05-1.43) | HRZM |  | |
| 1.10(0.40-3.01) | 0.70(0.18-2.82) | 0.52(0.12-2.32) | 1.90(0.69-5.28) | MRZE |  |
| 0.53(0.14-1.91) | 0.39(0.09-1.58) | 1.42(0.59-3.44) | 0.38(0.08-1.84) | 0.75(0.45-1.25) | HRZE |

(F) Death from all cause by the end of treatment

| HRZG |  | | |
| --- | --- | --- | --- |
| 0.85(0.19-3.82) | HRZM |  | |
| 0.82(0.20-3.27) | 0.96(0.18-5.08) | MRZE |  |
| 0.32(0.02-4.36) | 1.01(0.34-3.04) | 1.19(0.24-6.05) | HRZE |

(G) Death from all cause during intensive phase

| HRZELo |  | | | | |
| --- | --- | --- | --- | --- | --- |
| 1.73(0.13-23.90) | HRZO |  | | | |
| 5.29(0.17-169.22) | 3.06(0.12-76.64) | HRZG |  | | |
| 1.02(0.06-16.70) | 0.59(0.03-12.66) | 0.19(0.00-8.72) | HRZM |  | |
| 0.15(0.00-4.35) | 0.08(0.00-3.15) | 0.03(0.00-1.98) | 0.14 (0.00-6.06) | MRZE |  |
| 0.58(0.07-4.53) | 0.19(0.01-4.03) | 0.98(0.10-9.50) | 0.61(0.03-13.15) | 6.80(0.35-132.49) | HRZE |
