# Supplementary material for: Effects of Fluroquinolones in Newly Diagnosed, Sputum-Positive Tuberculosis Therapy: A Systematic Review and Network Meta-Analysis
Source: PLoS One. 2015 Dec 15;10(12):e0145066. doi: 10.1371/journal.pone.0145066 (PMC4682926; doi:10.1371/journal.pone.0145066)
Supplement: S5 Table — (DOC) [file pone.0145066.s006.doc]

**S5 Table. Evaluation of consistency using “loop specific” approach.**

| **Item** | **Loop** | **IF** | **CI_95** | **Loop_Heterog_tau^2^** |
| --- | --- | --- | --- | --- |
| Week-8 sputum negativity by LJ solid method | HRZE-HRZM-HRZO | 0.58 | 0.00-2.03 | 0.04 |
|  | HRZE-HRZG-HRZO | 0.42 | 0.00-1.60 | 0.00 |
|  | HRZE-HRZG-HRZM | 0.33 | 0.00-0.99 | 0.00 |
|  | HRZE-HRZM-MRZE | 0.29 | 0.00-0.89 | 0.02 |
|  | HRZG-HRZM-HRZO | 0.08 | 0.00-1.53 | 0.00 |
| Week-8 sputum negativity by liquid method | HRZE-HRZM-HRZO | 0.21 | 0.00-0.67 | 0.00 |
|  | HRZE-HRZM-MRZE | 0.06 | 0.00-1.31 | 0.00 |
|  | HRZE-HRZG-HRZM | 0.06 | 0.00-1.27 | 0.00 |
| Treatment failure by the end of treatment | HRZE-HRZG-HRZM | 1.16 | 0.00-3.86 | 0.00 |
|  | HRZE-HRZM-MRZE | 0.74 | 0.00-3.94 | 0.00 |
| Serious adverse events by the end of treatment | HRZE-HRZG-HRZO | 0.43 | 0.00-1.33 | 0.00 |
| Serious adverse events during intensive phase | HRZE-HRZM-HRZO | 0.96 | 0.00-3.08 | 0.00 |
|  | HRZE-HRZG-HRZM | 0.96 | 0.00-3.23 | 0.00 |
| Death from all cause by the end of treatment | HRZE-HRZM-MRZE | 0.08 | 0.00-2.04 | 0.00 |
| Death from all cause during intensive phase | Insufficient observations | | | |
|  | | | | |
