# Supplementary material for: Effects of Fluroquinolones in Newly Diagnosed, Sputum-Positive Tuberculosis Therapy: A Systematic Review and Network Meta-Analysis
Source: PLoS One. 2015 Dec 15;10(12):e0145066. doi: 10.1371/journal.pone.0145066 (PMC4682926; doi:10.1371/journal.pone.0145066)
Supplement: S6 Table — (DOC) [file pone.0145066.s007.doc]

**S6 Table. Assessment of global inconsistency in networks using the “design-by-treatment”** interaction model.

| **Network outcome** | **Chi-square** | **P value for test of global inconsistency** |
| --- | --- | --- |
| Week-8 sputum negativity by LJ solid method | 1.76 | 0.94 |
| Week-8 sputum negativity by liquid method | 1.09 | 0.58 |
| Treatment failure by the end of treatment | 1.36 | 0.72^*^ |
| Serious adverse events by the end of treatment | 1.15 | 0.56^*^ |
| Serious adverse events at the intensive phase | 1.43 | 0.23 |
| Death from all cause by the end of treatment | 0.73 | 0.70 |
| Death from all cause at the intensive phase | 0.10 | 0.75 |

^*^results calculated by fixed model.
