# Supplementary material for: Effects of Fluroquinolones in Newly Diagnosed, Sputum-Positive Tuberculosis Therapy: A Systematic Review and Network Meta-Analysis
Source: PLoS One. 2015 Dec 15;10(12):e0145066. doi: 10.1371/journal.pone.0145066 (PMC4682926; doi:10.1371/journal.pone.0145066)
Supplement: S1 Fig — (A) Treatment failure by the end of treatment; (B) Serious adverse events by the end of treatment; (C) Serious adverse events during intensive phase; (D) Death from all cause by the end of treatment; (E) Death from all cause during intensive phase. (DOC) [file pone.0145066.s008.doc]

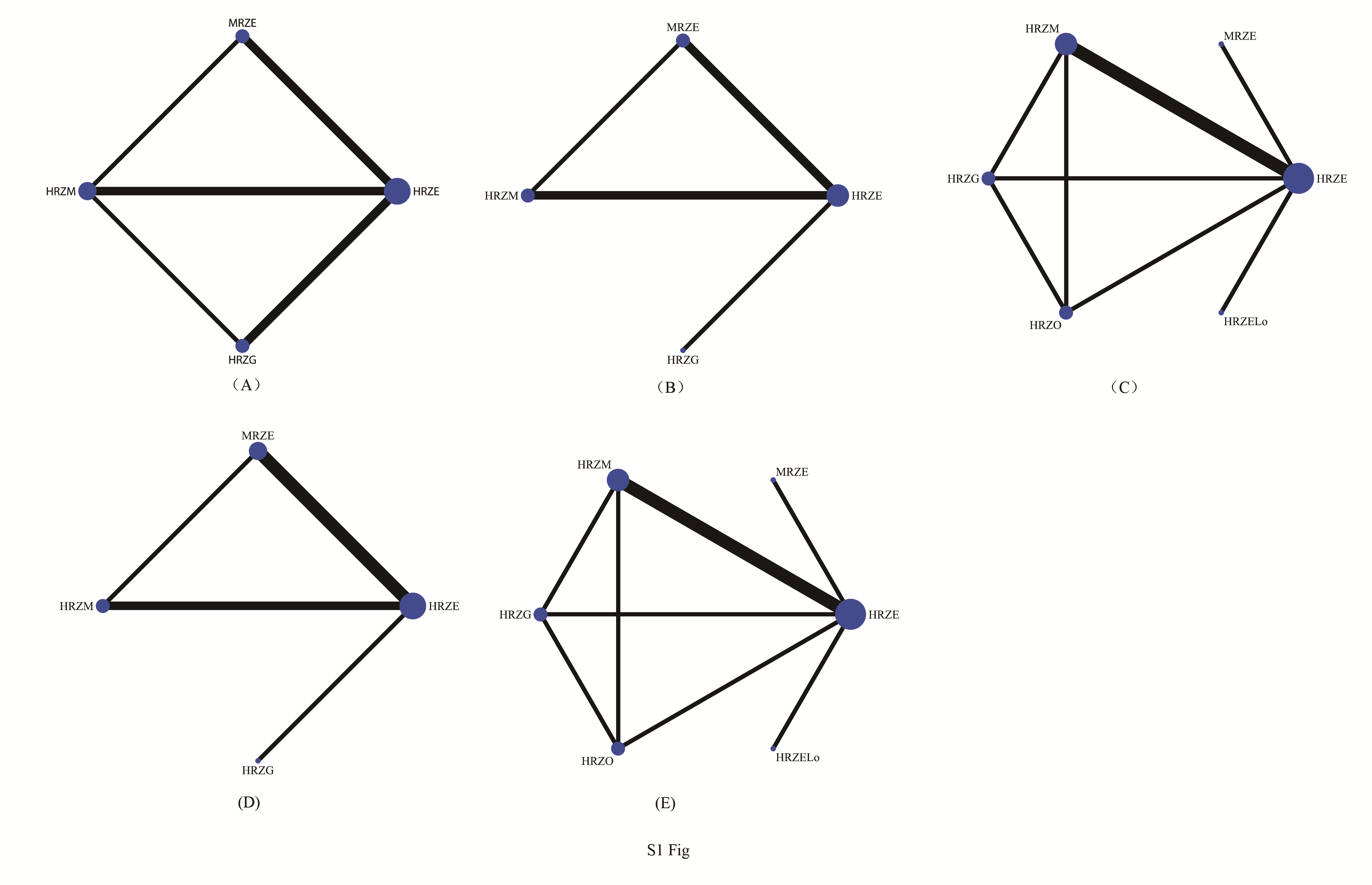


**S1 Fig. Network of possible interventions for second outcomes.** (A) Treatment failure by the end of treatment; (B) Serious adverse events by the end of treatment; (C) Serious adverse events during intensive phase; (D) Death from all cause by the end of treatment; (E) Death from all cause during intensive phase.
